# Soybean Oil Replacement by Poultry Fat in Broiler Diets: Performance, Nutrient Digestibility, Plasma Lipid Profile and Muscle Fatty Acids Content

**DOI:** 10.3390/ani11092609

**Published:** 2021-09-05

**Authors:** Ahmed A. Saleh, Abdulrahman S. Alharthi, Rashed A. Alhotan, Mustafa Shukry Atta, Abdel-Moneim Eid Abdel-Moneim

**Affiliations:** 1 Department of Poultry Production, Faculty of Agriculture, Kafrelsheikh University, Kafrelsheikh 33516, Egypt; ahmed.saleh1@agr.kfs.edu.eg; 2 Department of Animal Production, College of Food and Agriculture Sciences, King Saud University, Riyadh 11451, Saudi Arabia; Abalharthi@ksu.edu.sa (A.S.A.); ralhotan@ksu.edu.sa (R.A.A.); 3 Department of Physiology, Faculty of Veterinary Medicine, Kafrelsheikh University, Kafrelsheikh 33516, Egypt; 4 Nuclear Research Center, Biological Applications Department, Egyptian Atomic Energy Authority, Abu-Zaabal 13759, Egypt; aeabdelmoneim@gmail.com

**Keywords:** poultry fat, growth performance, plasma lipid, muscle FAs content, broilers

## Abstract

**Simple Summary:**

The effect of partial or complete substitution of soybean oil (SO) by poultry fat (PF) on growth, nutrient digestibility, plasma lipids, and the pectoral muscle content of fatty acids (FAs) was examined in this study. Dietary PF supplementation improved breast muscle FA profile but did not affect muscle vitamin E content and liver thiobarbituric acid reactive substances (TBARS). By adding PF to the diet, economic efficiency was greatly improved in a dose-dependent manner. Therefore, the results of this study revealed that PF could be used as a partial or total replacement of SO in broiler nutrition without affecting their performance or physiological response with a tendency to improve their meat products.

**Abstract:**

Continuous genetic improvements of commercial broiler strains has led to the necessity of using fats in their rations to fulfill a large portion of the energetic requirements. Several fat sources have been introduced in poultry nutrition, such as rendering poultry fat (PF) an available and cheap lipid source compared to conventional sources such as soybean oil (SO). The present study investigated the effect of partial or full replacement of SO by PF on performance, nutrient digestibility, blood lipids, and fatty acids (FAs) content of pectoral muscle. Four hundred and eighty one-day-old male Ross-308 chicks were distributed into four experimental groups (12 replicates each): the first group (control) was fed a diet formulated with soybean oil as a fat source while the second to fourth groups (PF25, PF50, and PF100) were fed diets formulated with 25, 50 and 100% of PF as a fat source instead of SO. Results revealed no synergistic effect between SO and PF in any of the studied parameters. Replacing SO by PF did not alter birds’ growth, carcass characteristics, and plasma indices of birds. Abdominal fat% was increased (*p* < 0.01) in PF50 and PF100. Dry matter digestibility was improved (*p* < 0.05) in PF50 and PF100, while crude fat and protein digestibility was not affected. Contents of palmitic and docosahexaenoic acids in the pectoral muscle of PF50 and PF100 were reduced (*p* < 0.01) while concentrations of oleic and linolenic acids, total unsaturated FAs, and polyunsaturated FAs/Saturated FAs ratio were elevated (*p* < 0.05) in the same groups. Liver thiobarbituric acid reactive substances (TBARS) and muscle vitamin E contents were not altered. The dietary addition of PF greatly improved economic parameters. In conclusion, PF can be used as a lipid source in broiler diets to produce inexpensive meat while maintaining its growth performance.

## 1. Introduction

The metabolizable energy requirements of modern commercial broiler strains are increasing with continuous improvements in their performance and the swift evolution of the intensive poultry industry. Additionally, due to the limited amount and incessant rising in prices of conventional energy sources feedstuffs, it has necessitated the search for alternative materials to ensure the future profitability of poultry production [1]. Lipid sources (oils and fats) are commonly included in broiler feeds to fulfill their high energy requirements. Fats added to broiler diets can improve the absorption and digestion of fat-soluble vitamins and other nutrients, enhancing their growth performance under armor neutral and heat stress conditions [2,3]. However, the prices of different lipid sources vary, and their utilization also varies depending on their physical and chemical properties [4]. The chain length of fatty acids (FAs) and their saturation degree affect the digestibility and metabolizable energy of the fat source [5]. Although animal fats are cheaper than vegetable oils, it is generally believed that the nutritional value of the former is less than that of the latter. Furthermore, among animal fats, poultry fat (PF) can be utilized by poultry species at a higher rate than tallow and lard oil [6].

In the Middle East, soybean oil (SO) is the most common fat source used in poultry diet formulation, particularly after several hydrations, filtration, and degumming [7]. However, limited supply and consistently higher prices are anticipated for it. Rendered poultry fats, obtained from processed wastes of poultry slaughterhouses, can be sustainable alternatives to SO due to their wide availability and relatively low price. Moreover, dietary addition of PF has other benefits, including reducing dustiness, improving feed texture, increasing palatability, and enhancing nutrient absorption by reducing digesta rate of passage through the gut. However, rancidity and low utilization, particularly in young chicks, are disadvantages of using animal fats in poultry diets [8]. No differences in performance parameters were noticed in the literature when PF was used instead of SO [9,10]. However, it’s worth noting that a synergistic effect was observed when vegetable oils were mixed with animal fats such as PF and tallow [6,11].

PF is commonly used in feed mills; however, the prohibitions and specific rules of the European Union regarding this practice have caused problems for these facilities with the EU countries [12,13,14]. Nevertheless, these by-products must be reintroduced into production and economy because of their high nutritional content, economic value, large production cost, and the high cost of alternative implementation. Therefore, the legal regulations and prohibitions put into action must be reconsidered to keep pace with the continuous improvement in production, use, and sales following current scientific developments.

The present study aimed to investigate the potential synergistic impact of mixing SO with PF in broiler diets as well as the effect of full replacement of SO by PF on performance parameters, nutrient digestibility, plasma lipids, and contents of fatty acids and α-tocopherol in pectoral muscle and TBARS in hepatic tissues.

## 2. Materials and Methods

### 2.1. Ethical Statement

The study was approved by the Ethics Committee of Local Experimental Animals Care Committee and conducted following the guidelines of Kafrelsheikh University, Egypt (Number 4/2016 EC). All precautions were followed to minimize suffering during the entire experimental period.

### 2.2. Chemical Analysis of Fat Sources

Poultry fat by-product and soybean oil were supplied by the Al-Sabeel Al-Gadidah Company (Tanta, Al-Gharbia, Egypt). Poultry fat was produced from the processed waste of poultry slaughterhouses (feathers, non-edible viscera, feet, head, blood, etc.) in a rendering unit with a batch-cooker and fat presser at the Fat Hanz Company (Tanta city, Egypt). Poultry fat was assessed for *Escherichia coli* and *Salmonella* spp., and it was incorporated into the diets only after confirming the absence of these pathogens. Metabolizable energy, peroxide value, and fatty acids profile of SO and PF were determined according to the procedures described by AOAC [15], Abd El-Moneim and Sabic [1], and Abd El-Moneim, et al. [16]. Values of the thrombogenic and atherogenic indexes were estimated following the equations of Ulbricht and Southgate [17]:

(1) $$Atherogenic\;index=\frac{C12:0+4*C14:0+C16:0}{Sum\;of\;unsaturated\;FAs}$$

(2) $$\mathrm{Thrombogenic}\;\mathrm{index}=\frac{C14:0+C16:0+C18:0}{0.5*\mathrm{OA}+0.5*\left(\mathrm{MUFA}-\mathrm{OA}\right)+0.5*n6\mathrm{PUFA}+3*n3\mathrm{PUFA}+\left(\frac{n3\mathrm{PUFA}}{n6\mathrm{PUFA}}\right)}$$

where OA = oleic acid (C18:1), MUFA = monounsaturated FAs, PUFA = polyunsaturated FAs.

### 2.3. Experimental Design

A total of 480 one-day-old male Ross-308 broiler chicks (43 g) were allocated into 48 ground pens and distributed equally into four experimental groups (12 replicates each). Pens, the stocking density of which was ten birds/m², were equipped with an automatic nipple cup drinker and a chain feeder system. The starter (0–10 d), grower (11–24 d), and finisher (25–35 d) experimental diets (Table 1) were formulated to meet the recommendation of Aviagen [18] for male broilers. 

The first diet (basal diet; control) was formulated using soybean oil as a fat source. In the second to fourth experimental diets, 25, 50, and 100% of the soybean oil were replaced by poultry fat. Diets and freshwater were offered ad libitum to the birds. The feed trial took place in a temperature-controlled chamber, started from 33 ± 1 °C and decreased by one degree per 3 days until reaching 24 ± 1 °C and kept till 35 days of age, with a proportional humidity between 50% and 70% and a 22:2 h light: dark cycle. Mortalities were recorded throughout the experimental phases.

### 2.4. Growth Performance and Organ Weights

Initial and final body weight and feed consumption were measured individually on a pen basis. Feed conversion ratio (FCR) was calculated as a g feed:g gain. The European production efficiency factor (EPEF) was calculated as liveability (%) × body weight (g)/FCR × age (d). At the end of the trial (35 days), 48 birds (one bird/replicate; 12 birds/treatment) were individually weighed, slaughtered by cutting the carotid artery, and dissected to evaluate the relative weights of the thigh and breast muscles, abdominal fat, and liver [19]. Blood samples were collected with heparinized test tubes for blood biochemical analyses.

### 2.5. Nutrient Digestibility

At the end of the experiment (35 d), the twelve birds per group were weighed and individually caged in metabolic pens for collecting their excreta for four days. Before the commencement of this period, an adaptation period of 24 h had elapsed. Fresh water and diets were offered ad libitum to all birds during the manure collection stage. The approximate analysis of dry matter (#930.15), crude protein (#954.01), and crude fat (#920.29) of dried excreta and diets were performed according to AOAC [15]. The trichloroacetic acid procedure was used to estimate fecal nitrogen [20].

### 2.6. Plasma Biochemical Analysis

At 35 days, to separate plasma, collected blood samples were centrifuged (2500× *g* for 15 min at 4 °C). Plasma samples were kept at −20 °C pending analysis. Plasma high-density lipoprotein (HDL), total cholesterol, aspartate aminotransferase (AST), total protein, and albumin were calorimetrically evaluated using commercial kits and following the manufacturer’s instructions (Diamond Diagnostics, Cairo city, Egypt), using spectrophotometric analysis (Spectronic 1201; Milton Roy, Ivyland, PA, USA).

### 2.7. Muscle and Liver Biochemical Analysis

The analysis of superficial pectoral muscle FAs was conducted on 48 birds (1 bird per replicate; 12 birds per treatment) using gas-liquid chromatography (GLC) as described by [21,22]. The concentrations of muscle vitamin E [23] and liver thiobarbituric acid-reactive substances (TBARS) [24] were also determined.

### 2.8. Economic Efficiency

Average feed cost per bird was calculated as feed consumption per bird × cost of one kg diet (0.53, 0.52, 0.51 and 0.50 US $ for control, PF25, PF50, and PF100, respectively, considering the price of one kg of SO (1.47 US $) and PF (0.32 US $). Feed cost per kg gain was estimated by multiplying the cost of a one kg diet by the FCR. Total costs were measured by summing the feed cost/bird and all fixed costs, including housing labor, vaccines, drugs, day-old chick, disinfectant, veterinary supervision, etc. Subtracting total costs from the total return, considering the average price of the bird (1.72 US $ per one kg live body weight), was considered the net return. Benefit/cost ratio (B/C ratio) was estimated by the following equation: net return/total costs × 100 [25].

### 2.9. Statistical Analysis

Differences between the experimental groups were analyzed using one-way ANOVA was applied to determine the effects of replacing SO with PF, in which pens were the statistical units for performance parameters, birds for the carcass, organ weights, and samples for biochemical and other parameters, the General Linear Model package of SPSS (Version 19.0, Chicago, IL, USA). Tukey’s multiple range test was used to identify the significant (*p* < 0.05) differences among means of experimental groups.

## 3. Results

### 3.1. Chemical Analysis of Fat Sources

The main difference between the FAs profile of SO and PF (Table 2) can be found in the ratio of unsaturated FAs over-saturated FAs (U/S ratio). The U/S ratio of SO (3.84) was 1.7 times that of PF (2.28). Concentrations of polyunsaturated (PU) FAs in SO were higher than that of PF, while the latter was richer in monounsaturated (MU) FAs. The PU/S ratio of SO was 2.4 times that of PF, while the MU/PU ratio of PF was 2.2 times that of SO.

### 3.2. Growth Performance and Organ Weights

The partial or full replacement of SO by PF did not alter the final body weight, feed consumption, and FCR of broiler chickens at marketing age (Table 3). The EPEF of PF groups was slightly higher than that of the control. The highest value of EPEF was recorded in PF100. Additionally, the dietary inclusion of different lipid sources had no significant impact on carcass percentage and the relative weight of thigh and breast muscles and liver of 35-day-old broiler chicks. The abdominal fat percentage was increased (*p* < 0.01) in PF100 and PF50 compared to control and PF25.

### 3.3. Nutrient Digestibility

As presented in Table 4, digestibility coefficients of dry matter were improved (*p* < 0.01) in PF50 and PF100 compared to the control. Digestibility coefficients of crude protein and fat were not influenced by dietary replacement of PF instead of SO.

### 3.4. Plasma Biochemical Analysis

The data presented in Table 5 shows the impact of partial or total replacement of SO by PF on plasma biochemical parameters of broilers at marketing age. Total protein, albumin, AST, HDL-cholesterol, and total cholesterol were not significantly affected by dietary inclusion of PF compared to the control.

### 3.5. Muscle and Liver Biochemical Analysis

The fatty acid profile of the pectoral muscle was influenced by the type of dietary fat source (Table 6). The main differences in the FAs profile of breast muscle can be found in the concentrations of certain FAs and the PU/S ratio. Concentrations of palmitic acid and docosahexaenoic acids were reduced (*p* < 0.01) in PF50 and PF100. However, levels of oleic acid, linolenic acid, total unsaturated FAs, and PU/S ratio were elevated (*p* < 0.05) in the pectoral muscle of birds fed 50% and 100% PF. Pectoral muscle concentration of α-tocopherol and content of TBARS in the liver were not significantly affected by replacing SO with PF (Table 6). However, numerical reduction in α-tocopherol and elevation in TBARS levels in PF treated groups were observed.

### 3.6. Economic Efficiency

As presented in Table 7, economic parameters were greatly influenced by partial and full replacement of SO by PF in broiler diets. Feed cost/bird, feed cost/kg gain, and total cost/bird were decreased (*p* < 0.001) in PF25, PF50, and PF100 compared to the control. Net return and benefit/cost ratio were increased (*p* < 0.001) in PF25, PF50, and PF100, and the highest values were recorded in group PF100.

## 4. Discussion

The analyzed composition of the lipids sources in the present study revealed that PUFAs in SO were higher than that of PF while the MUFAs in PF were higher. The U/S and PU/S ratios of SO were 1.7 and 2.4 times that of PF, while the MU/PU ratio of PF was 2.2 times that of SO. These findings are almost similar to NRC [26] and previous findings [8,27]. The predominant FA in PF was oleic acid followed by linoleic acid, while in SO, it was linoleic acid followed by oleic acid. This revealed that the two fat sources were rich in the unsaturated FAs with the superiority of SO by 12.5%, which may explain the effects of these lipids on studied parameters.

Fats are a high-energy feedstuff commonly incorporated in the formulation of commercial poultry diets. Results of earlier studies investigating the impact of dietary addition of different lipid sources on poultry performance were equivocal. Several investigations have reported that supplementation of vegetable oils to poultry feed can improve their performance, carcass traits, and production efficiency by elevating the diet’s energy level better than animal fat [28,29,30]. Others reported a synergistic effect between vegetable oils and animal fat [6,11]. Nevertheless, some studies revealed non-significant differences between animal fat and vegetable oils [9,10,27]. In the present study, no synergy effect between PF and SO was noticed. We also found that partial and total replacement of SO by PF in broiler diets had no significant impact on final body weight, FCR, EFEF, and carcass traits except abdominal fat, which increased PF50 and PF100. A similar trend was reported by Okur [31] and Sanz [32], who noticed an elevation in abdominal fat weight when animal fats were used in broiler diets. The growth performance of broilers is greatly influenced by dietary fat sources and their FA profiles, particularly essential FAs, such as α-linolenic acid and linoleic acid, as their deficiency may retard broilers growth [33]. As our results revealed, the differences between SO and PF in these FAs were insufficient to induce significant differences in birds’ growth performance. The lack of differences in growth performance of birds fed diets with SO or PF could also be attributed to the equilibrium ratios of energy-to-protein and energy-to-amino acid in these diets [27,31]. Additionally, Pesti et al. [27] reported that feeding on fat sources with high metabolizable energy resulted in a high amount of fat being deposited. This might explain the increase of abdominal fat in birds fed diets with high levels of PF.

In our study, replacing SO by PF improved dry matter digestibility while digestibility of crude protein and crude fat was not affected. These results are in line with previous findings [34,35]. Fatty acids chain length and U/S ratio greatly affect nutrient digestibility. Fat sources such as tallow and palm oil characterized by a low U/S ratio showed drastic negative impacts on nutrients digestibility [36,37]. Tancharoenrat et al. [35] noticed a reduction in crude fat digestibility for broilers fed diets with fat sources with low U/S ratio such as palm oil (U/S 0.93) and tallow (U/S 0.80) compared to SO (U/S 5.07).

In the present study, the difference between the U/S ratio of PF (2.28) and SO (3.84) was lower than mentioned in the study of Tancharoenrat et al. [35], which might explain the insignificant changes in fat and protein digestibility. Nevertheless, they did not observe a significant difference for crude fat digestibility between PF- (U/S 2.07) and SO-based diets. The authors suggested that the changes in FAs composition of PF were not enough to exert a drastic effect on crude fat digestibility. Furthermore, dietary addition of PF could enhance nutrients’ digestion and absorption by reducing digesta rate of passage through the gut, which might explain the improvement in dry matter digestibility [8].

To our knowledge, limited investigations have studied the impact of dietary addition of fat sources on the blood biochemistry of poultry species. The majority of these studies focused on the impact of fat types on the quality of animal products for human uses without studying their effect on birds’ health status during production [38]. In the present study, we evaluated the effect of replacing SO by PF on broiler chickens’ hepatic function and blood lipids. No significant alterations were observed in all studied parameters among experimental groups. Hu [9] noticed similar results who reported insignificant impact of dietary SO and PF on HDL levels- and LDL-cholesterol and total cholesterol in the serum of Cherry Valley ducks. Donaldson et al. [38] also noticed non-significant changes in serum levels of AST, total protein, albumin, and cholesterol of Japanese quail-fed diets with SO and lard. However, results of serum triglycerides as affected by various dietary fat sources and levels were somewhat contradictory. Some studies reported significant elevated serum triglycerides of humans and birds fed high dietary fat [39,40]. Others reported non-significant changes [9,41], while some observed a significant reduction in its level [38,42]. Donaldson et a. [38] attributed the decrease in serum triglycerides to a possible reduction in de novo synthesis of FAs in the liver as large amounts of FAs were being supplied to the birds via dietary fat sources. The lack of consistency between findings of these studies suggests some differences in lipid handling pathways and multiple potential mechanisms contribute to regulating serum concentrations of cholesterol and triglycerides between different avian species, including postabsorptive lipid metabolism and/or hepatic uptake of HDL-cholesterol.

Consumers have become more concerned about the nutritional aspects, including the lipid profile and FA contents. Chicken meat with its low-fat and high-protein contents has been characterized as the main source of PUFAs [43]. Functional and beneficial foods that contribute to preventing chronic diseases, such as coronary heart disease and metabolic disorders, are characterized by higher concentrations of PUFAs [44,45]. It has been documented that FAs and lipid profiles of chicken meat can be modified by changing broilers’ feed composition [46,47]. Reducing SFAs and elevation of PUFAs contents in chicken meat would improve its nutritional value and quality [48]. Our results showed that pectoral muscle levels of palmitic acid and docosahexaenoic acids decreased while concentrations of oleic acid, linolenic acid, total UFAs, and PU/S ratio were elevated PF50 and PF100. These findings are considered positive as the reduction in PU/S ratio, thrombogenic index, and the atherogenic index, and the elevation of linolenic acid (n-3) are favorable in healthy and functional food for human consumption. The added value of n-3 PUFAs to human foods and their favorable impacts of on human health were investigated. Bostami et al. [47] reported the health benefits of long-chain n-3 FAs to animals and humans, such as reducing the risk of heart diseases and lowering the concentration of circulating cholesterol.

Moreover, Pinchasov and Nir [49] documented that PUFAs can inhibit the activity of the 9-desaturase enzyme complex, which responsible for converting SFAs to MUFAs, thereby downregulating the synthesis of MUFAs. Furthermore, the reduction in the atherogenic and thrombogenic indexes in PF50 and PF100 is considered favorable, as Ulbricht and Southgate [17] recommended the low values of these indexes in healthy human diets. Generally, supplementation of PF in broiler diets instead of SO tends to improve the lipid profile of breast meat.

Lipid oxidation, one of the major factors responsible for the deterioration of the quality of meat products, is primarily initiated in the UFAs of membrane phospholipids [17]. Vitamin E (α-tocopherol) plays a fundamental role in protecting these susceptible cellular structures against oxygen-containing free radicals and reduces their content of TBARS [50]. The primary location of vitamin E is within the biological membranes, such as mitochondria and microsomes, which allow its effective function compared to other antioxidants [17]. Therefore, increasing the muscle membrane content of α-tocopherol by dietary manipulation is required. Dietary fat sources generally contain high fat-soluble vitamins, including α-tocopherol, but they vary among themselves. Vegetable oils and most plant-origin feedstuffs are rich in vitamin E, while their content is lower than most animal products [51,52,53,54]. In the present study, levels of α-tocopherol in pectoral muscle and TBARS in the liver did not differ by replacing SO with PF. Numerical reduction in α-tocopherol and elevation in TBARS levels in PF treated groups were observed. These findings agree with those of Polycarpo et al. [55], who reported significant changes in hepatic contents of vitamins E and A (fat-soluble vitamins) of broilers fed on corn-based diets with SO or beef tallow as lipid sources. Lauridsen, et al. [56] also noticed that fat sources did not influence the concentration of vitamin E in muscle membranes. Contrarily, Dänicke, et al. [56] and Gatellier, et al. [57] observed higher hepatic vitamin A and muscular TBARS concentrations in birds fed diets with SO compared with tallow. The lack of significance in α-tocopherol concentrations in the present study might be attributed to the low incorporation levels of SO and PF in broilers diet, eliminating the added value of vitamin E to the feed.

Furthermore, the insignificant differences in fat digestibility observed in this study may be considered another explanation as absorption of fat-soluble vitamins depends on fat digestibility and the emulsification process. Knarreborg, et al. [58] documented that good conditions of micelle formation and emulsion increase the bioavailability of α-tocopherol. The numerical reduction in α-tocopherol and increase in TBARS concentrations in the breast and liver tissues of birds fed on PF may be due to the relatively high susceptibility of broiler meat to lipid oxidation when fed diets incorporated with PF [59,60].

As expected, the economic efficiency and benefit-to-cost ratio were significantly improved by dietary replacement of SO by PF in a dose-dependent manner. This effect is due to the large difference between the prices of SO and PF; under our study’s condition, the price of SO was 4.6 times that of PF. The differences in the prices of SO and PF are reasonable; since SO is one of the most commonly used fat sources in poultry rations and its various industrial uses. While PF is a cheap by-product of poultry slaughterhouses, its utilization is affordable and reduces its adverse impacts on the environment. Several studies have reported the economic benefits of using PF instead of SO [4,9,34,38].

## 5. Conclusions

The present study investigated the effect of partial or full replacement of SO by PF on growth performance, nutrient digestibility, plasma lipids, and vitamin E and FAs contents of the pectoral muscle of broilers. Neither a synergistic effect between SO and PF nor effects of the dietary changes on broilers’ growth, carcass parts, and blood biochemistry were noticed in the present study. Dietary supplementation of PF improved the FA profile of breast muscle but did not affect muscle content of vitamin E and liver TBARS levels. Economic efficiency was greatly improved in a dose-dependent manner by dietary addition of PF. Therefore, this study revealed that PF can be used as a partial or total replacement of SO in broilers’ nutrition without affecting their performance or physiological response to improve their meat products.

## Figures and Tables

**Table 1:** Composition of the experimental starter (1–11 d), grower (11–24 d), and finisher (25–35 d) diets.

Ingredient, g/kg	Control			PF25			PF50			PF100		
	Starter	Grower	Finisher	Starter	Grower	Finisher	Starter	Grower	Finisher	Starter	Grower	Finisher
Yellow corn	536	586	646	533	585	645	535	585	644	534	582	641
Soybean meal, 46%	358	302	225	364	307	231	369	313	237	377	323	250
Corn gluten meal, 62%	40	42	55	37	38	50	30	32	45	23	25	35
Soybean oil	24.00	29.00	32.00	18.00	21.75	24.00	12.00	14.50	16.00	0.00	0.00	0.00
Poultry Fat	0.00	0.00	0.00	6.00	7.25	8.00	12.00	14.50	16.00	24.00	29.00	32.00
Dicalcium phosphate	16	15	15	16	15	15	16	15	15	16	15	15
Dl Methionine, 99%	2.1	2.0	1.4	2.1	2.0	1.4	2.1	2.0	1.4	2.1	2.0	1.4
l-Lysine HCl, 98%	3.4	3.7	4.6	3.4	3.7	4.6	3.4	3.7	4.6	3.4	3.7	4.6
l-Threonine	0.5	0.3	0.1	0.5	0.3	0.1	0.5	0.3	0.1	0.5	0.3	0.1
CaCo3	12	12	11	12	12	11	12	12	11	12	12	11
NaCl	3.5	3.5	3.5	3.5	3.5	3.5	3.5	3.5	3.5	3.5	3.5	3.5
Premix *	3	3	3	3	3	3	3	3	3	3	3	3
NaCo3	1.5	1.5	1.6	1.5	1.5	1.6	1.5	1.5	1.6	1.5	1.5	1.6
K2Co3	0.0	0.0	1.8	0.0	0.0	1.8	0.0	0.0	1.8	0.0	0.0	1.8
	Chemical Analysis											
Crude protein, %	23.22	21.14	19.00	23.23	21.14	19.01	23.21	21.15	19.02	23.21	21.15	19.02
AME kcal/kg	2967	3060	3165	2967	3061	3166	2967	3062	3167	2968	3062	3167
Ca, %	0.950	0.896	0.864	0.950	0.895	0.864	0.950	0.897	0.864	0.960	0.894	0.864
Available P, %	0.422	0.408	0.388	0.422	0.408	0.388	0.422	0.408	0.388	0.422	0.408	0.388
Crude fiber, %	3.444	3.547	3.333	3.786	3.992	3.909	4.311	4.327	4.334	5.444	5.131	5.509
Na, %	0.193	0.193	0.196	0.193	0.193	0.196	0.193	0.193	0.196	0.193	0.193	0.196
Cl, %	0.250	0.250	0.250	0.250	0.250	0.249	0.250	0.254	0.249	0.250	0.248	0.249

PF25, 25% of soybean oil replaced by PF; PF50, 50% of soybean oil replaced by PF; PF100, full replacement of soybean oil by PF. Apparent metabolizable energy (*AME*); Calcium (Ca); Available Phosphorus (Available P)l Sodium (Na); Chloride (Cl). * Composition of Hero mix^®^ premix. (per 1 kg): Vitamin A 4,000,000 IU, vitamin E 3334 mg, vitamin D3 833,000 IU, vitamin B1 250 mg, vitamin K3 500 mg, vitamin B6 500 mg, vitamin B2 1250 mg, vitamin B12 3.33 mg, folic acid 333.4 mg, biotin 16.7 mg, niacin 10,000 mg, pantothenic acid 3334 mg, iron 10,000 mg, zinc 16,700 mg, manganese 20,000 mg, selenium 33.4 mg, iodine 100 mg, copper 1334 mg, and cobalt 33.4 mg).

**Table 2:** Fatty acid profile and energy values of soybean oil and poultry fat.

Item	Soybean Oil	Poultry Fat
Myristic acid (C14:0), %	0.01	0.47
Palmitic acid (C16:0), %	14.69	21.83
Palmitoleic acid (C16:1), %	-	3.14
Heptadecanoic acid (C17:0), %	-	0.20
Stearic acid (C18:0), %	5.50	7.59
Oleic acid (C18:1 n-9), %	26.80	32.06
Vaccenic acid (C18:1 n-7), %	-	1.79
Octadecanedioic acid (C18:2 n-4), %	-	0.24
Linoleic acid (C18:2 n-6), %	44.90	29.26
Linolenic acid (C18:3 n-3), %	6.95	1.69
Arachidic acid (C20:0), %	0.35	0.20
Gadoleic acid (C20:1 n-9),%	0.31	0.20
Eicosatrienoic acid (C20:3 n-3), %	-	0.20
Arachidonic acid (C20:4 n-6), %	-	0.55
Non identified fatty acids	0.49	0.58
Saturated fatty acids	20.55	30.29
Unsaturated fatty acids	78.96	69.13
Monounsaturated fatty acids	27.11	37.19
Polyunsaturated fatty acids	51.85	31.94
Unsaturated fatty acids/Saturated fatty acids	3.842	2.282
Polyunsaturated fatty acids/Saturated fatty acids	2.523	1.054
Monounsaturated fatty acids/Polyunsaturated fatty acids	0.523	1.164
Atherogenic index	0.187	0.343
Thrombogenic index	0.354	0.762
Peroxide value, meq/kg	1.85	4.26
Gross energy, kcal/kg	8400	9460

Milliequivalents per kilogram (mEq/kg); Kilocalorie per Kilogram (kcal/kg).

**Table 3:** Effect of replacing soybean oil with poultry fat on growth performance and organ weights of broilers.

Item	Experimental Diets ^1^				SEM ^2^	*p*-Value
	Control	PF25	PF50	PF100		
Initial body weight, g	43.11	43.00	43.20	43.02	0.032	0.896
Body weight 35 d, g	2131.7	2142.1	2148.3	2157.5	10.33	0.851
Feed consumption 35 d, g	3340.4	3335.0	3330.8	3324.6	10.04	0.957
FCR, g feed:g gain	1.60	1.59	1.58	1.57	0.041	0.188
Mortality, %	0.833	0.833	0.833	0.833	0.089	0.894
EPEF	377.6	382.2	384.9	388.9	3.228	0.664
	Organ weights, %					
Carcass	67.22	67.41	67.72	67.91	0.172	0.497
Breast muscle	23.13	23.27	23.43	23.62	4.243	0.279
Thigh muscle	16.52	16.54	16.22	16.32	0.068	0.262
Liver	2.24	2.28	2.14	2.31	0.028	0.152
Abdominal fat	1.31 ^b^	1.47 ^b^	1.90 ^a^	1.93 ^a^	0.055	0.002

^1^ PF25, 25% of soybean oil replaced by PF; PF50, 50% of soybean oil replaced by PF; PF100, full replacement of soybean oil by PF. ^2^ SEM, standard error of means. EPEF, European production efficiency factor; FCR, feed conversion ratio. ^a,b^ Means within the same row with different superscripts differ. Gram (g).

**Table 4:** Effect of replacing soybean oil with poultry fat on nutrient digestibility of broilers.

Item	Experimental Diets ^1^				SEM ^2^	*p*-Value
	Control	PF25	PF50	PF100		
Dry matter, %	71.14 ^b^	72.57 ^ab^	74.27 ^a^	74.80 ^a^	0.491	0.022
Nitrogen, %	69.21	69.93	70.11	70.32	0.264	0.506
Fat, %	77.89	79.93	80.65	80.73	0.392	0.053

^1^ PF25, 25% of soybean oil replaced by PF; PF50, 50% of soybean oil replaced by PF; PF100, full replacement of soybean oil by PF. ^2^ SEM, standard error of means. ^a,b^ Means within the same row with different superscripts differ.

**Table 5:** Effect of replacing soybean oil with poultry fat on plasma biochemical parameters of broilers.

Item	Experimental Diets ^1^				SEM ^2^	*p*-Value
	Control	PF25	PF50	PF100		
**Aspartate aminotransferase, mg/dL**	232.3	225.4	234.9	221.2	3.960	0.607
**Total protein, mg/dL**	3.72	3.58	3.57	3.77	0.053	0.457
**Albumin, mg/dL**	2.05	2.03	2.11	2.13	0.038	0.743
**Total cholesterol, mg/dL**	160.2	157.0	146.3	149.3	2.441	0.155
**HDL-cholesterol, mg/dL**	80.17	81.00	84.75	85.83	1.071	0.169

^1^ PF25, 25% of soybean oil replaced by PF; PF50, 50% of soybean oil replaced by PF; PF100, full replacement of soybean oil by PF. ^2^ SEM, standard error of means. High Density lipoprotein (HDL-cholesterol); milligrams per decilitre (mg/dL).

**Table 6:** Effect of replacing soybean oil with poultry fat on fatty acids profile of broilers pectoral muscle.

Item	Experimental Diets ^1^				SEM ^2^	*p*-Value
	Control	PF25	PF50	PF100		
Myristic acid (C14:0), %	1.39	1.38	1.38	1.37	0.035	0.981
Palmitic acid (C16:0), %	22.12 ^a^	21.07 ^ab^	20.05 ^b^	20.02 ^b^	0.270	0.008
Palmitoleic acid (C16:1), %	5.46	5.41	5.49	5.43	0.175	0.977
Stearic acid (C18:0), %	9.01	9.17	9.07	9.03	0.189	0.953
Oleic acid (C18:1 n-9c), %	41.55 ^b^	43.23 ^ab^	44.01 ^a^	44.06 ^a^	0.329	0.012
Vaccenic acid (C18:1 n-7), %	5.22	5.45	5.48	5.08	0.160	0.807
Linoleic acid (C18:2 n-6), %	9.37	9.26	9.48	9.89	0.132	0.363
Linolenic acid (ALA, C18:3 n-3), %	0.66 ^b^	0.64 ^b^	0.78 ^a^	0.88 ^a^	0.027	0.001
Arachidonic acid (AA, C20:4 n-6), %	2.32	2.89	2.56	2.62	0.104	0.304
Eicosapentaenoic acid (EPA, C20:5 n-3), %	0.064	0.068	0.062	0.064	0.002	0.811
Docosapentaenoic acid (DPA, C22:5n-3), %	0.32	0.319	0.317	0.318	0.009	0.916
Docosahexaenoic acid (DHA, C22:6n-3),%	0.979 ^a^	0.990 ^a^	0.809 ^b^	0.872 ^b^	0.022	0.002
Saturated fatty acids	32.53	31.62	30.51	30.43	0.356	0.109
Unsaturated fatty acids	65.94 ^b^	68.24 ^ab^	68.99 ^a^	69.22 ^a^	0.510	0.049
Monounsaturated fatty acids	52.23	54.08	54.99	54.57	0.438	0.114
Polyunsaturated fatty acids	13.71	14.16	14.00	14.65	0.154	0.182
Unsaturated fatty acids/Saturated fatty acids	2.032 ^b^	2.160 ^ab^	2.271 ^a^	2.275 ^a^	0.031	0.006
Polyunsaturated fatty acids/Saturated fatty acids	0.423 ^b^	0.448 ^ab^	0.461 ^a^	0.482 ^a^	0.007	0.027
Monounsaturated fatty acids/Polyunsaturated fatty acids	3.810	3.822	3.932	3.744	0.040	0.431
Atherogenic index	0.420 ^a^	0.390 ^b^	0.371 ^b^	0.369 ^b^	0.006	0.001
Thrombogenic index	0.853 ^a^	0.804 ^ab^	0.772 ^b^	0.759 ^b^	0.011	0.006
Vitamin E, mg/100 g muscle	0.327	0.312	0.288	0.285	0.067	0.224
Liver TBARS, nmol/g	18.33	18.67	19.50	21.50	0.478	0.126

^1^ PF25, 25% of soybean oil replaced by PF; PF50, 50% of soybean oil replaced by PF; PF100, full replacement of soybean oil by PF. ^2^ SEM, standard error of means. ^a,b^ Means within the same row with different superscripts differ. ThioBarbituric Acid Reactive Substances (*TBARS*); milligrams per 100 g (mg/100 g).

**Table 7:** Effect of replacing soybean oil with poultry fat on the economic parameters.

Item	Experimental Diets ^1^				SEM ^2^	*p*-Value
	Control	PF25	PF50	PF100		
Feed cost/bird, US $	1.770 ^a^	1.734 ^b^	1.699 ^c^	1.662 ^d^	0.01	<0.001
Feed cost/kg gain, US $	0.848 ^a^	0.827 ^b^	0.807 ^c^	0.787 ^d^	0.01	<0.001
Total cost, US $/bird	2.951 ^a^	2.890 ^b^	2.831 ^c^	2.771 ^d^	0.01	<0.001
Total return, US $/bird	3.592	3.610	3.621	3.637	0.02	0.850
Net return, US $/bird	0.642 ^c^	0.720 ^b^	0.790 ^b^	0.866 ^a^	0.03	<0.001
B/C ratio, %	21.73 ^d^	24.89 ^c^	27.90 ^b^	31.21 ^a^	0.61	<0.001

^1^ PF25, 25% of soybean oil replaced by PF; PF50, 50% of soybean oil replaced by PF; PF100, full replacement of soybean oil by PF; B/C, benefit/cost. ^2^ SEM, standard error of means. ^a–d^ Means within the same row with different superscripts differ.

## Data Availability

Data are available upon request.
